# Editorial: Investigating the role of extracellular vesicles in cardiovascular disease mechanisms

**DOI:** 10.3389/fcvm.2026.1994562

**Published:** 2026-10-02

**Authors:** Jona Benjamin Krohn, Elena Aikawa

**Affiliations:** 1 Deutsches Herzzentrum der Charité, Department of Cardiology, Angiology and Intensive Care Medicine, Berlin, Germany; 2 Charité - Universitätsmedizin Berlin, Freie Universität Berlin and Humboldt-Universität zu Berlin, Berlin, Germany; 3 Center for Interdisciplinary Cardiovascular Sciences, Department of Medicine, Brigham and Women’s Hospital, Harvard Medical School, Boston, MA, United States; 4 Center for Excellence in Vascular Biology, Department of Medicine, Brigham and Women’s Hospital, Harvard Medical School, Boston, MA, United States

**Keywords:** acute myocardial infarction, atherosclerosis, cardiovascular disease, extracellular vesicles, vascular calcification

As key mediators of local and distant intercellular communication, extracellular vesicles (EVs) have emerged as an increasingly important focus in cardiovascular disease research in recent years (1). Tissue-derived EVs, and particularly circulatory EVs, have emerged as a rich source of biomarkers that remains largely inaccessible to current diagnostic approaches. Analysis of the molecular EV cargo offers a unique window into the state of their cells of origin, enabling a form of liquid biopsy for organs and tissues that are difficult to access directly, such as the heart and vasculature. On the therapeutic side, engineering EVs enriched with a specific cargo to target a distinct cell population poses a promising tool to deliver cell- or tissue-specific remedies. The evidence provided in this research topic presents a comprehensive overview of late-breaking studies exploring all aforementioned facets of EVs in cardiovascular disease.

Delving into the spectrum of cardiovascular tissue-derived EV populations, Bheri et al. identified parent and recipient cell-specific preferences in EV uptake mechanisms, ranging from caveolae- and lipid raft-dependent endocytosis, clathrin-mediated uptake, and micropinocytosis. Using cardiac progenitor cells (CPC), mesenchymal stem cells (MSC) or stromal cells, the authors argue that parent cell type determines lipid composition, and, surprisingly, to a much lesser extent, proteome of their EV progeny. Thus, their study directs the focus toward the EV lipidome and EV membrane composition as currently understudied categories of EV cargo. The authors further identified multiple novel EV membrane surface targets, among those MRC2, which is hypothetically implicated in MSC/CPC-derived EV uptake by endothelial cells via a clathrin-mediated mechanism to impact endothelial tube formation.

With a particular focus on blood-derived EV, Bernath-Nagy et al. highlighted current evidence on this easily accessible biomarker compartment with respect to its diagnostic, prognostic and therapeutic potential. In acute coronary syndrome, EV-enriched marker proteins, RNA, and distinct phospholipid composition of the EV membrane allow for the reliable discrimination from healthy individuals and those with stable coronary artery disease. A comparable diagnostic accuracy is ascribed to the blood-derived EV transcriptome and proteome in atrial fibrillation. Moreover, blood-derived EV-enriched cargo may aide in the diagnosis of fulminant myocarditis or post-heart transplant allograft rejection, thus potentially rendering invasive and complication-prone endomyocardial biopsies obsolete. The authors further highlighted the relative abundance of platelet- and leucocyte-derived EV and EV-specific cargo as prognostic markers of adverse cardiovascular events in different patient cohorts. Herein, distinct transcriptional and translational EV signatures were found to be predictive of adverse LV remodeling as a harbinger of cardiovascular mortality. Regarding the therapeutic potential of EV, evidence exploring the effect of conditioned or patient blood-derived EV in *in vitro* or *in vivo* models uniformly pointed towards a beneficial effect of EV on the inflammatory response, angiogenesis, and endothelial activation in cardiovascular tissue remodeling. In terms of prognostic ability, blood-derived EV signatures in patients under certain medications such as anti-Xa-inhibitors, antithrombotic agents, statins or antiarrhythmic drugs underscored the diagnostic potential of circulating EV to assess treatment response. Furthermore, blood-derived EVs and their molecular cargo could predict outcomes following TAVR, in patients on VA-ECMO, or in high-risk patients following carotid endarterectomy or percutaneous coronary intervention.

In an effort to translate mechanistic studies on EV-tissue interactions to the organismal level whilst limiting experimental complexity, Koutentaki et al. elaborated on the living myocardial slice (LMS) model, a bioreactor involving a slice of heart tissue to maintain tissue integrity in culture, to investigate the role of EV in cardiovascular tissues. LMS can be exploited for functional analyses encompassing contractility studies, live calcium imaging, and conduction velocity measurements in an *in vitro* setting. To this end, the LMS model can be applied in two major analytical directions: first, the effect of different conditions and/or stressors on heart tissue in terms of global quantitative or qualitative EV secretion can be interrogated. Second, the effect of specific EV on cardiac tissue- and/or cell type-specific transcriptional signatures can be investigated to identify EV-mediated disease networks that can potentially be leveraged for EV-based targeted therapies.

Exploring the potential of EV-based therapies, Fu et al. provided a summary of evidence on engineered EVs as a mode of delivery in cardiovascular disease contexts. Herein, stem cell and progenitor cell-derived EVs were shown to exert beneficial effects through immunomodulation, activation of reparatory processes, or angiogenesis. Moreover, EV multiomics approaches have been shown to enable biomarker discovery, while subsequent findings may identify mechanisms for selectively targeting cardiovascular tissues using EV with a distinct surface integrin composition or through conjugation with homing peptides. To further the notion of a therapeutic modulation of detrimental EV release, Patel et al. investigated the effect of epigallocatechin gallate (EGCG), a senolytic agent found in green tea, on the senescence-associated secretory phenotype (SASP) in endothelial cells (EC) commonly associated with proinflammatory EV release and EV-mediated monocyte activation. EGCG treatment of senescent EC resulted in the secretion of EV capable of reversing cytokine secretion by THP-1 cells in response to senescent EC-EV and lipopolysaccharide stimulation.

As technical advances allow for the increasingly resource-efficient purification of EV from various biological materials, EVs offer a rare insight into the state of a cell or tissue and have therefore become an attractive target in cardiovascular research. Through the identification of EV-bound molecules of interest, and translation of these findings into clinically applicable assays, EVs assume a new frontier in translational medicine, potentially shaping a future generation of diagnostic methods to identify, stratify, and treat cardiovascular disease. Joint efforts should address the current heterogeneity of EV purification methods, and aim to develop accessible platforms for EV content and surface modulation for the development of targeted, cell-specific molecular therapies. As universal messengers of health and disease, EVs offer immense potential in a field in growing demand for widely applicable, causative therapies.
